# Androgen promotes differentiation of PLZF^+^ spermatogonia pool via indirect regulatory pattern

**DOI:** 10.1186/s12964-019-0369-8

**Published:** 2019-05-29

**Authors:** Jingjing Wang, Jinmei Li, Wei Xu, Qin Xia, Yunzhao Gu, Weixiang Song, Xiaoyu Zhang, Yang Yang, Wei Wang, Hua Li, Kang Zou

**Affiliations:** 1 Germline Stem Cells and Microenvironment Lab, College of Animal Science and Technology, Nanjing Agricultural University, Weigang NO.1, Xuanwu District, Nanjing, 210095 China; 2 Bio-ID Center, School of Biomedical Engineering, Shanghai Jiao Tong University, Shanghai, China; 3 National Experimental Teaching Demonstration Center of Animal Science, Nanjing Agricultural University, Nanjing, 210095 China

**Keywords:** Androgen receptor, β1-integrin, PLZF, Spermatogonial stem cells, WT1

## Abstract

**Background:**

Androgen plays a pivotal role in spermatogenesis, accompanying a question how androgen acts on germ cells in testis since germ cells lack of androgen receptors (AR). Promyelocytic leukemia zinc-finger (PLZF) is essential for maintenance of undifferentiated spermatogonia population which is terminologically called spermatogonia progenitor cells (SPCs).

**Aims:**

We aim to figure out the molecular connections between androgen and fates of PLZF^+^ SPCs population.

**Method:**

Immunohistochemistry was conducted to confirm that postnatal testicular germ cells lacked endogenous AR. Subsequently, total cells were isolated from 5 dpp (day post partum) mouse testes, and dihydrotestosterone (DHT) and/or bicalutamide treatment manifested that *Plzf* was indirectly regulated by androgen. Then, Sertoli cells were purified to screen downstream targets of AR using ChIP-seq, and gene silence and overexpression were used to attest these interactions in Sertoli cells or SPCs-Sertoli cells co-culture system. Finally, these connections were further verified in vivo using androgen pharmacological deprivation mouse model.

**Results:**

Gata2 is identified as a target of AR, and β1-integrin is a target of Wilms’ tumor 1 (WT1) in Sertoli cells. Androgen signal negatively regulate β1-integrin on Sertoli cells via Gata2 and WT1, and β1-integrin on Sertoli cells interacts with E-cadherin on SPCs to regulate SPCs fates.

**Conclusion:**

Androgen promotes differentiation of PLZF^+^ spermatogonia pool via indirect regulatory pattern.

**Electronic supplementary material:**

The online version of this article (10.1186/s12964-019-0369-8) contains supplementary material, which is available to authorized users.

## Plain English summary

Androgen signaling plays a pivotal role in spermatogenesis, but the molecular mechanisms underlying androgen action in this process are unclear. Specifically, it is unknown if androgen receptor (AR) is expressed in germ cells. Thus, it’s interesting to reveal how androgen induces differentiation of spermatogonial stem cells (SSCs). Here we observed the AR is deficient in SSCs before spermatogenesis onset. Then we examined a regulatory role of the AR in spermatogenesis using a SSCs-Sertoli cells co-culture system, and demonstrated that androgen negatively regulated the key gene for SSCs stemness, *Plzf*. Additionally, we identified Gata2 as a target of AR in Sertoli cells, and demonstrated that Wilms tumor 1 (WT1) and β1-integrin as two putative intermediate molecules to transfer differentiation signals from Sertoli cells to SSCs, which was further verified using androgen pharmacological-deprivation mice model. These results demonstrate a regulatory pattern of androgen in SSCs niche in an indirect way via multiple steps of signal transduction: androgen activates AR in Sertoli cells to indirectly regulate ITGB1 on Sertoli cells via intermediate molecule GATA2 and WT-1, and ITGB1 on Sertoli cells interacts with E-cadherin on SSCs to regulate SSCs fates.

## Background

Spermatogonial stem cells (SSCs) reside in the basement membrane and are subjected to control signal in the microenvironment to function as the original source for sperms in male testis. In the hierarchy of differentiation, spermatogonia consist of different subpopulations such as A_s_, A_pr_, A_al_, A_1_…A_4_, A_In_, B etc. Of them, A_s_, A_pr_ and A_al_ spermatogonia are referred to as undifferentiated population, and are nominated as spermatogonial progenitor cells [1]. The mechanism of SSCs differentiation is not clear yet, especially the role of androgen in spermatogenesis is still a mystery. Androgen is produced by testicular Leydig cells and functions via binding and activation of its receptor AR in cytoplasm. Activated AR is subsequently imported into nucleus after release of heat shock protein and functions as a transcription factor [2]. Numerous target genes of AR have been identified, involving in many biological processes such as gonad development [3] and tumorigenesis [4]. However, the exact biological mechanism of AR in spermatogenesis is not fully understood, and whether AR is expressed in testicular germ cells is controversial. Some studies detected AR signal in germ cells of mouse testis [5, 6]. Other studies suggested that AR is presented only in the somatic cells of rodent testis [7]. It has been shown that germ cell specific *Ar* knockout mice still had normal sperm [8] but conditional deletion of AR in Leydig or Sertoli cells caused spermatogenesis defects [9, 10]. These results suggest that AR expressed in Sertoli cells, Leydig cells and perivascular myoid cells may participate in spermatogenesis via interacting with surrounding spermatogonia[11]. However, Sycp1-driven Cre for *Ar* deletion in germ cells was used in the study mentioned above[8], which only indicates AR is not required in germ cells since meiosis onset. Moreover, studies reported that androgen functions as a signal molecule in SSCs niche, namely androgen acts on peritubular myoid (PM) cells surrounding the seminiferous tubule to stimulate PM cells to produce GDNF, to promote self-renewal of SSCs [12, 13], indicating a complicated role of androgen in testicular niche. In all, the mechanism of spermatogenesis mediated by androgen still needs to be further investigated.

*Plzf* is a key transcription suppressor gene for SPCs maintenance. It was first discovered by its association with acute promyelocytic leukemia [14], and was subsequently characterized as an undifferentiated marker for SSCs in rodents[15] and primates [16]. Loss of *Plzf* did not affect spermatogonia formation, but led to progressive and significant deficiency of SSCs after neonatal life and finally caused infertility [15, 17], indicating its critical role in SSCs maintenance. Moreover, PLZF expression was detected in spermatogonia A_s_, A_pr_ and A_al_, not restricted in SSCs [18]. Thus, PLZF is a marker of SPCs, and PLZF is an important factor for maintenance of this pool [19]. Although the link of androgen and PLZF has not been reported in germ cells, much evidence from prostate tumorigenesis studies suggests the interaction between AR and PLZF. For example, *Plzf* represses prostate tumorigenesis and its expression can be inhibited by androgen antagonist, bicalutamide [20]. In prostate cancer cell line PCa cells, PLZF was identified as a repressor of AR as well as an activator of regulated in development and DNA damage responses 1 (REDD1), which suppressed mTORC1 [21]. AR was characterized as a critical transcriptional factor in prostate tumorigenesis [4], and mTORC1 has been found to participate in EMT (Epithelial mesenchymal transition) in prostate cancer [22]. Thus, PLZF functions as tumor suppressor and interacts with AR in prostate cancer system, but it’s unclear whether similar links exist in germ line.

In testis, Sertoli cells in base membrane form niches to protect SSCs and regulate their fates [23], and many surface proteins, such as cadherins and integrins, are identified as functional components in the niche [24]. Many of these molecules are AR responsive and associated with the fate of SSCs [25], but the mechanism is largely unknown. Also, it’s necessary to focus on *Wt-1* gene, which is specifically expressed in Sertoli cells and required for Sertoli cell lineage maintenance [26, 27]. Moreover, WT1 functions as a suppressor of *Ar* [28]. Thus, we ask whether WT1 participates in the regulation of spermatogenesis mediated by androgen signal.

Here, we studied AR expression pattern in testis of postnatal mouse using a monoclonal antibody, and detected weak AR signal in pre-spermatogonia of 2 dpp testes, but found that this signal was absent in germ cells from 3 dpp, instead appeared exclusively in somatic cells. Spermatogenesis starts from about 5 dpp [29], so the possibility that germ cells need AR for spermatogenesis is eliminated. Thus, we investigated the indirect regulation pattern of androgen on SPCs differentiation via Sertoli cells using a SPCs-Sertoli cells co-culture system. Two transcription factors Gata2 and WT1 were identified as AR’s downstream targets in Sertoli cells, and a surface protein β1-integrin was characterized as a target of WT1 and may function as an intermediate molecule to transfer androgen signal from Sertoli cells to SPCs to regulate spermatogenesis. In all, we demonstrates the complicated regulation pattern of androgen in spermatogenesis in SSCs niche, which requires the cooperation of Sertoli cells and many intermediate molecules to regulate *Plzf* expression in undifferentiated spermatogonia populations.

## Methods

### Animals

CD-1 mice were supplied by Comparative Medicine Centre of Yangzhou University, and all animal experiments have been approved by Animal Protection Committee of Nanjing Agricultural University.

### Isolation and culture of total testicular cells or SPCs

Total testicular cells extracted from testes of 5 dpp mice followed previous protocol [30] with minor modifications. Testes were cut into small particles and followed by collagenase IV and trypsin digestion. Undigested collagen was removed using micropippetor after centrifugation, and cell pellet was resuspended in culture medium and then transferred to gelatin coated plates for culture. To identify the total testicular cells’ co-culture system, the expression of *Sox9* and *Wt1* (Sertoli cells marker), *Coup-tf II* (Leydig cells marker), *Sma* (Peritubular Myoid cells marker), *Mvh* (germline marker), *Ar*, *Scp3* (meiosis marker), *c-kit* (SPCs differentiation marker), *Integrin-αV*, *Cd9*, *Integrin-β1 (itgb1)*, *Integrin-α6* and *Plzf* (SPCs marker) were detected using RT-PCR (Fig. 2u and v).

For SPCs sorting, resuspended cells after two-enzyme digestion were filtered with 70-μm filter and subsequently incubated with mouse Thy-1.2 antibody coated magnetic beads (BD, Cat.551518). Thy1^+^ fraction was collected and cultured on mitotically inactivated STO feeder layer at 37 °C with 5% CO_2_. Culture medium for total testicular cells or SPCs was composed of 90% MEMa, 10% FBS (Gibco), 1 ng/ml bFGF (Sino Bio Inc., 10,014-HNAE), 1 ng/ml EGF (Sino Bio Inc., 50,482-MNAY), 1 ng/ml GDNF (Sino Bio Inc., 10,561-HNCH), 10 ng/ml LIF (Santa Cruz, sc-4378), 20 μg/ml transferrin (sigma, T8158), and 5 μg/ml insulin (Aladdin, I113907). For androgen stimulation or deprivation, dihydrotestosterone (sigma, cat. 31,573) or bicalutamide (sigma, PHR-1678) was added to the culture medium for 48 h. To make feeder layers, STO cells were incubated with 10 μg/ml mitomycin C (Roche) at 37 °C with 5% CO_2_ for 2 h and plated on gelatin coated plates.

### Sertoli cell isolation

A modified method was used to isolate Sertoli cells from testes of 5 dpp mice [31]. Briefly, testes were encapsulated and seminiferous tubules were pooled and digested with 0.5 mg/mL collagenase IV. After centrifugation, the supernatant containing the Leydig cells was pipetted out. The tubules were further digested with 1 mg/mL collagenase IV and 10 μg/mL DNase to detach peritubular cells. Digestion was monitored under microscopy. The supernatant containing peritubular cells after centrifugation was discarded. Finally, single cell suspension was obtained after trypsin digestion and blocked by FBS. The cell pellet was resuspended in DMEM/F12 media supplemented with 2.5% FBS, 5% horse serum, 2 mM L-Gln, 100 U/ml penicillin, 1uM sodium pyruvate, and then plated. After 48 h, hypotonic treatment was performed to remove germ cells as described [32], and the purity of Sertoli cell was assessed by morphology and immunofluorescent staining of WT1 (purity of Sertoli cells> 95%) [Fig. 4a-d and i].

### Semi-quantitative RT-PCR (SqRT-PCR) and quantitative RT-PCR (qRT-PCR)

Total RNA was isolated using TRIzol (TIANGEN, DP405) and the first-strand cDNA was synthesized using a PrimeScript RT Master Mix (Takara, RR036A), SqRT-PCR was performed using Taq DNA polymerase (Takara, R001WZ), qRT-PCR was performed using TB Green premix Ex Taq II(Takara, RR820A) according to the instruction, the results were analyzed by 2−^△△Ct^ method and gene relative expression levels were normalized to *Gapdh* expression. The information of primers was listed in Additional file 1: Table S1.

### ChIP-seq and ChIP-qPCR

To characterize genome-wide binding patterns of AR and WT1 in Sertoli cells, ChIP and input DNA libraries were performed as previously described [33]. Briefly, cells were cross-linked with 1% formaldehyde for 10 min at room temperature and formaldehyde was then inactivated by the addition of 125 mM glycine for 5 min. Sonicated DNA fragments with 100–300 bp were pre-cleared and then immunoprecipitated with Protein A + G Magnetic beads coupled with anti-Androgen Receptor antibody (Millipore, 06–680) or anti-Wilms’ tumor 1 antibody (Abcam, ab89901). After reverse crosslinking, immunoprecipitated DNAs and input DNAs were end-repaired and ligated adapters to the DNA fragments using NEBNext Ultra End-Repair/dA-Tailing Module (E7442, NEB) and NEBNext Ultra Ligation Module (E7445, NEB). High-throughput sequencing of the ChIP fragments was performed using Illumina NextSeq 500 following the manufacturer’s protocols. The raw sequencing data were processed with trimmomatic (version 0.36) to filter low-quality reads [34]. The resulting data were mapped using bowtie2 (version 2.2.9) to the UCSC mm10 genome reference [35]. Peak detection was performed using the MACS peak finding algorithm (Model-based Analysis of ChIP-Seq; version 1.4.2) with parameters --nomodel --shiftsize 25 and the *p*-value cutoff set to 0.05 [36].

### Primers for ChIP-qPCR

GATA2-F: GCTAGAGAGTGCATTGGGGA GATA2-R: AGTTCCTGGGGCTGCGAG.

ITGB1-F: AGCTACATTTCTGAGCAATTATGGA ITGB1-R: CTGCTCTCCCAGGACTCAAC.

Control-F:TTAGGTGGCCTCAGATCCTC Control-R:CCTGCCTCTCTTTTGGACAG.

### Bicalutamide injection

Bicalutamide injection was performed as described [37] with minor modifications: 6-week old CD1 male mice were intraperitoneally injected with bicalutamide (20 mg/kg) or DMSO (vehicle) once every other day for 4 times.

### Immunohistochemistry (IHC) and immunofluorescence (IF)

Mouse testes were harvested and fixed in 4% neutral paraformaldehyde overnight, and subsequently dehydrated and embedded in paraffin. Histological sections were dewaxed and rehydrated in ethanol series, followed by microwave antigen retrieval in 0.01 M citrate (pH = 6.0) and methanol/H_2_O_2_ treatment at room temperature. After blocking with 5% goat serum, the slides were incubated with primary antibodies against PLZF (Santa Cruz, sc-22,839), AR (Abcam, ab133273), ITGB1 (Abcam, ab179471), c-Kit (Cell Signaling, 3074) and biotin labelled goat anti-mouse IgG (Vector, BA-9200) or goat anti-rabbit IgG (Vector, BA-1000), respectively. Streptividin-HRP (Jackson Lab) and DAB kit (Vector, sk4100) were used for visualization. Cell IF was carried out as described [38], primary antibodies against MVH (Cell Signaling, 8761 s), PLZF (Santa Cruz, sc-2831 and sc-22,839), WT1 (Abcam, ab89901), CDH1 (Abcam, ab76055), CD9 (Affinity, DF6565), ITGB1 (BD, 610467) and secondary antibodies Alexa Flour532 goat anti-rabbit (Invitrogen, A-11009), goat anti-mouse IgG-FITC (ZSGB-BIO, ZF-0312) are used.

### BrdU assay

Mice were intraperitoneally injected with BrdU (sigma, B5002, 50 mg/kg) 4 h before testis harvesting. Testes were fixed in 4% neutral PFA and embedded with paraffin. For BrdU detection, sections were incubated with 2 M HCl at 37 °C for 1 h, and washed with 0.1 M boric acid (pH = 8.5) for 3 times. IF was performed as aforementioned (BrdU, Santa Cruz, sc-32,323).

### Gene silence and overexpression

The validated siRNA against PLZF (5′-CCAGCAAGAUGUUUGAGAUTT-3′)[39], ITGB1 (5′-UAGAAAUGUUGGAACACUUUCGUCC-3′)[40],WT1 (5′-GGCGAUUGUUAAAGCUCAUUU-3′) [41], gata2 (5′-CGGGCACCUGUUGUGCAAAUUGUCA-3′) [42], control (5′-UUCUCCGAACGUGUCACGUTT-3′) and shRNA sequences against ITGB1 (5′-AATTCAAAAAAGCACGATGTGATGATTTAGAATCTCTTGAATTCTAAATCATCACATCGTGCG-3′)[43],control (5′-AATTCAAAAAACAACAAGATGAAGAGCACCAATCTCTTGAATTGGTGCTCTTCATCTTGTTGG-3′) were synthesized by Shanghai Genepharma Company. AR expression plasmid was a gift from Prof. Zijie Sun (Stanford University). Wt1 expression plasmid (EX-Mm24822–02) was purchased from GeneCopoeia. Plasmids or siRNA oligos were transfected using lipofectamine 3000 (Invitrogen, L3000008).

### Western blot and co-immunoprecipitation

For western blotting, tissue or cell lysates were separated in SDS PAGE gel and transferred to nitrocellulose membrane, and was subsequently blocked with 5% skim milk at 4 °C overnight and then incubated with primary antibodies Sycp3 (Santa Cruz, sc-74,569), Stra8 (Abcam, ab49602), claudin11 (Abcam, ab175236), nectin2 (Abcam, ab135246), GATA2 (Abclonal, A0677), GFRα1 (Affinity, DF7309), β-catenin (Cell Signaling, 8480p), β-tubulin (Anbo, P07437) and others mentioned before at room temperature for 1 h. The membranes were incubated with HRP-conjugated goat anti rabbit or mouse IgG (Santa Cruz, sc-2004,sc-2005) and ECL, and finally exposed the film. For Co-IP, lysates of adult mice testes or SPCs were prepared using a standard protocol (Beyotime, P0013), and 0.5 mg total proteins diluted with TBST were incubated with mouse anti-ITGB1 antibody (BD, 610467) and rabbit anti-CDH1 antibody (Santa Cruz, sc-7870) respectively and rocked at 4 °C overnight. The pre-washed protein (A + G) sepharose beads (Beytime, P2012) were added afterwards and incubated at 4 °C for 4 h. The beads were then washed three times with TBST, pelleted and boiled in 1XSDS loading buffer, and finally analyzed by western blot.

### Statistical analysis

For cell counting, sections or immunofluorescent visual fields were selected randomly. Data was analyzed by SPSS statistics 17.0 and was presented as mean ± SD (standard deviation). Statistical significance was determined by *t*-test.

## Results

### PLZF^+^ spermatogonia pool keeps steady during testis development

**Fig. 1:**
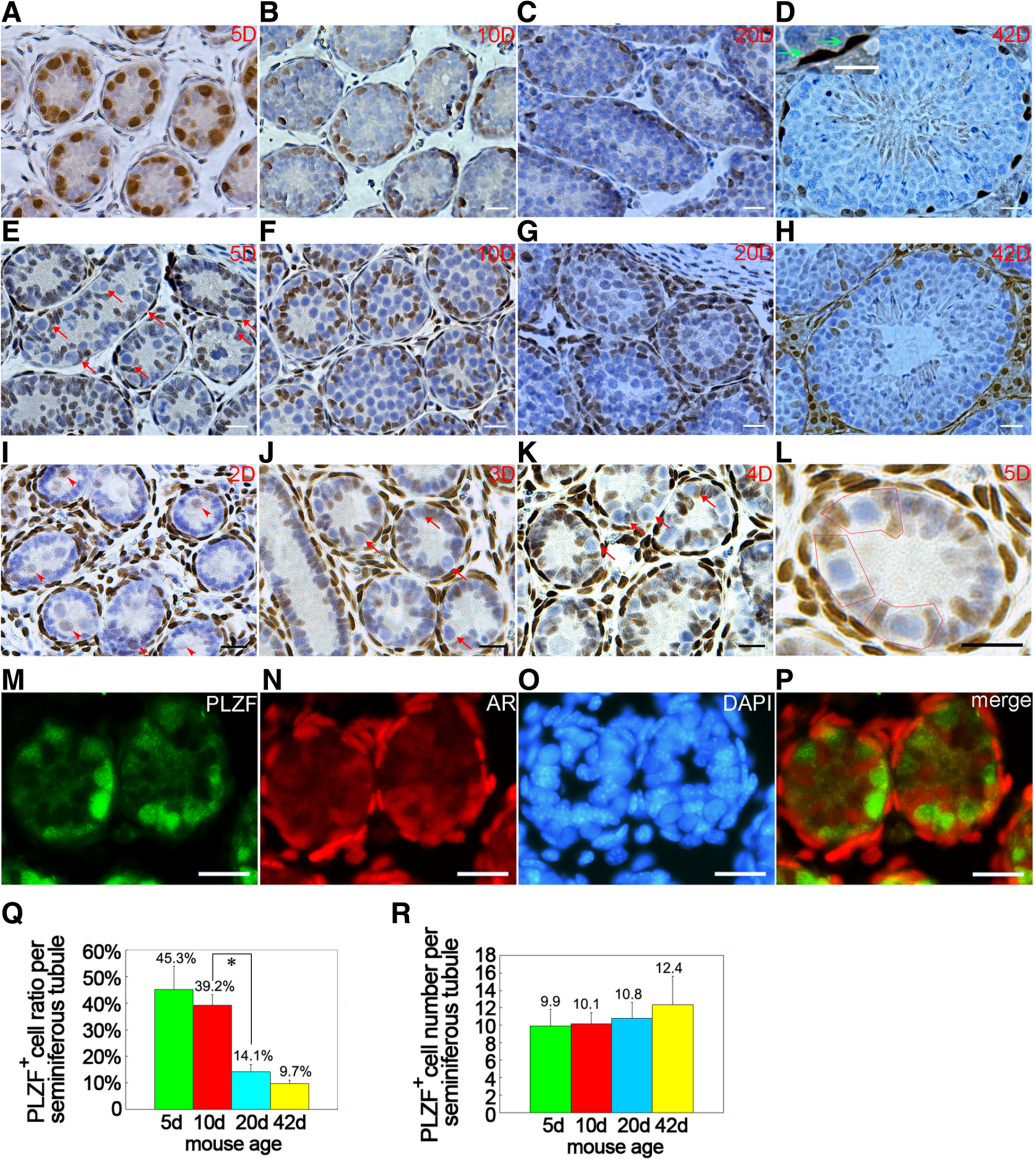
Expression patterns of PLZF and AR during testis development. PLZF staining was exclusively detected in spermatogonia localized in basal membranes of 5 dpp (**a**), 10 dpp (**b**), 20 dpp (**c**) and 42 dpp (**d**) testes (inset in D, a representive of PLZF^+^ A_pr_ spermatogonia). AR staining in 5 dpp (**e**), 10 dpp (**f**), 20 dpp (**g**) and 42 dpp (**h**) testes was specifically detected in Sertoli cells. Weak AR staining was observed in pre-spermatogonia (red arrowheads) in the testicular lumen of 2 dpp testis, but not in Sertoli cells (**i**). In 3 dpp testis (**j**), pre-spermatogonia migrated to basement membranes and formed unique structures with surrounding Sertoli cells (red arrows in (**j**)), and identical structures were also found in 4 dpp and 5 dpp testes, red arrows in (**e**) and (**k**)), and AR staining was detected in Sertoli cells but not in germ cells. **k** AR staining in 4 dpp testis, **l** a representive of niche in 5 dpp testis, in which SPCs were embraced by AR^+^ Sertoli cells (red frame). AR/PLZF dual IF exhibited no overlap of AR signals and PLZF signals in 4 dpp testis ((**m**). PLZF, (**n**). AR, (**o**). DAPI, (**p**). merge). From neonatal to adult stages, ratio of PLZF^+^ cells in seminiferous tubules decreased (**q**) but number of PLZF^+^ cells kept steady (**r**). Data represent means ± SD (**p < 0.01), *n* = 16, 4 of each group, bar = 20 μM

The size of PLZF^+^ population in testis represents the capacity of self-renewal and spermatogenesis, thus the expression profile of PLZF was detected in neonatal (5 dpp), juvenile (10 dpp), puberty (20 dpp) and adult (42 dpp) testes using IHC staining. The results demonstrated that from neonatal to adult testes, all PLZF^+^ cells exclusively located at basement membrane with strong nucleus staining [Fig. 1a-d], or a few oblate pairs of cells in adult testes, which are A_pr_ spermatogonia [Fig. 1d inset]. These PLZF^+^ cells were surrounded by Sertoli cells, which formed the microenvironment called niches. Strong PLZF intensity in 5 dpp testis indicated high expression level of PLZF at this stage. In testes from 5 to 42 dpp, the percentage of PLZF^+^ cells in seminiferous tubules was declined [Fig. 1q] due to continuous production of differentiated germ cells. However, the total number of PLZF^+^ cells was approximately consistent [Fig. 1r], indicating the pool of undifferentiated spermatogonia is stable during testicular development.

### AR expression in testis indicates an indirect regulation pattern in promoting spermatogenesis

Subsequently, we examined AR expression in 5, 10, 20 and 42 dpp testes using IHC, to investigate the role of androgen in spermatogenesis. Intensive AR signals were detected in Sertoli cells, Leydig cells and myoid cells but not in germ cells [Fig. 1e-h], consistent with the conclusion that postnatal testicular germ cells lacked endogenous AR [7]. In 5 dpp testes, moderate AR staining was detected in Sertoli cells localized at basement membrane [Fig. 1e]. Both AR^+^ Sertoli cells number and AR signal intensity were obviously increased in 10 dpp testes, and some AR^+^ Sertoli cells moved to the second layers [Fig. 1f], and then formed AR^+^ layers in 20 dpp testes [Fig. 1g]. In 42 dpp testis, those AR^+^ second layers disappeared and AR^+^ cells distributed in different layers of seminiferous tubules [Fig. 1h]. In rodent Sertoli cells, AR expression was reported to start from 3 to 5 dpp [44], thereupon we examined AR expression in 2, 3 and 4 dpp testes, and detected AR signal in Sertoli cells only from 3 dpp testis and signal intensity grew stronger from 5 dpp [Fig. 1I-k]. We also noticed Sertoli cells did not express AR in 2 dpp testis as literature reported [44], but unexpectedly observed weak AR signal in pre-spermatogonia localized in the cord of testes [Fig. 1I]. Interestingly, AR signal was absent in the pre-spermatogonia of 3 dpp testes, at which time point some of these pre-spermatogonia had already migrated from cord to basal membrane [Fig. 1j]. Meanwhile, Sertoli cells began to express AR and two AR^+^ Sertoli cells embraced one pre-spermatogonium to form a special structure nominated as ‘niche’ [Fig. 1j-l], implying AR in Sertoli cells commenced to function in microenvironment. Spermatogenesis starts from 3 to 5 dpp and differentiated spermatogonia can be observed at 6 dpp [29]. Dual immunofluorescent staining also exhibited no overlap of AR and PLZF signals in 4 dpp testes [Fig. 1m-p]. Collectively, AR expression pattern in Sertoli cells demonstrates their close connection with PLZF^+^ cells, but PLZF^+^ population is unable to be directly stimulated by androgen since they lack of endogenous AR when spermatogenesis starts.

### Blockage of androgen with antagonist inhibits SPCs differentiation

**Fig. 2:**
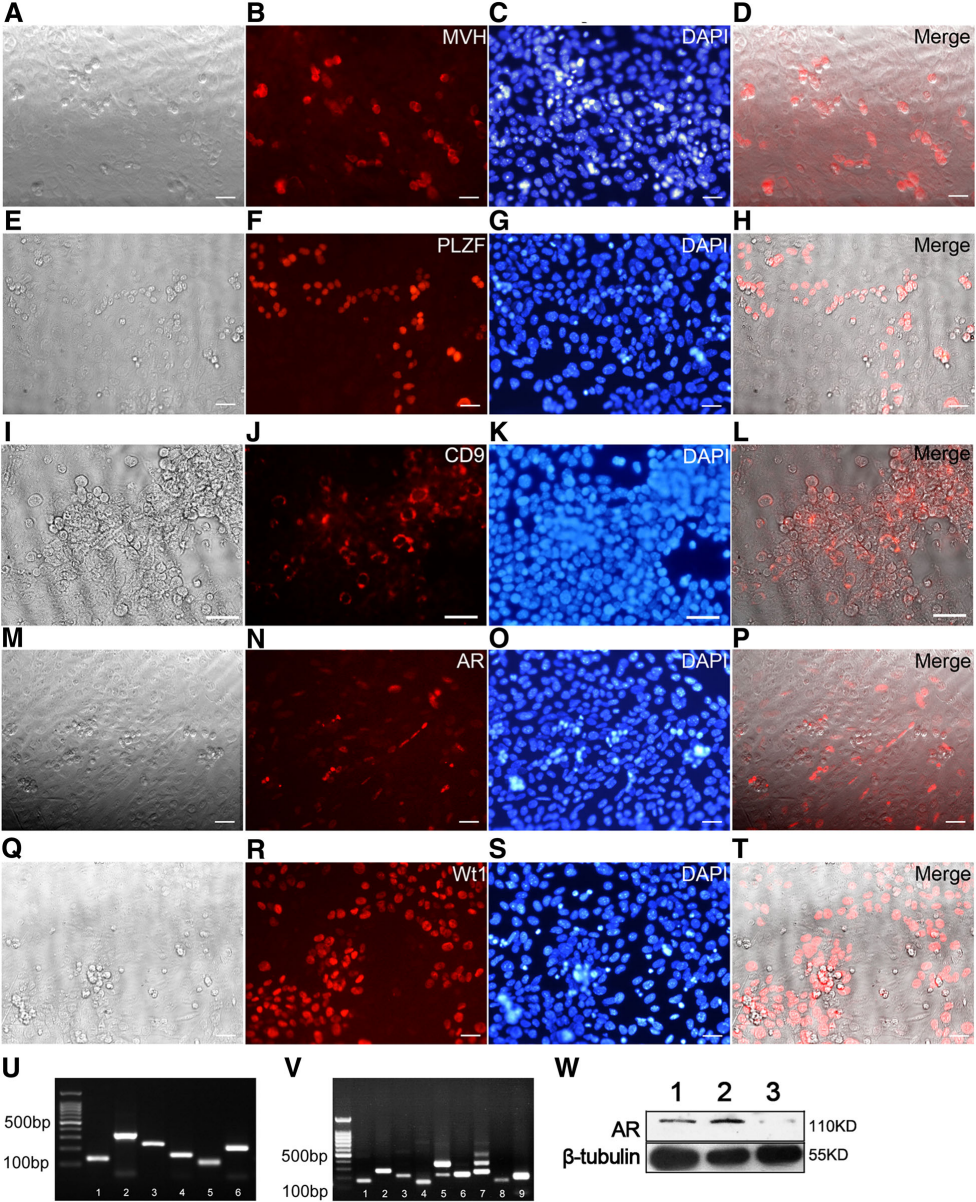
SPCs-somatic cells co-culture system was able to be maintained in vitro for short term. SPCs-somatic cells co-culture system stably expressed MVH ((**a**) bright filed, (**b**) MVH, (**c**) dapi, (**d**) merge), PLZF ((**e**) bright filed, (**f**) PLZF, (**g**) dapi, (**h**) merge), CD9 (**i**) bright filed, (**j**) WT1, (**k**) dapi, (**l**) merge), AR ((**m**) bright filed, (**n**) AR, (**o**) dapi, (**p**) merge) and WT1 ((**q**) bright filed, (**r**) WT1, (**s**) dapi, (**t**) merge). RT-PCR (*n* = 2) results exhibit the co-culture system expresses somatic markers ((**u**) 1.*Gapdh*, 2.*Sox9*, 3. *Sma*, 4.*Coup-tf II*, 5. *Wt1*, 6.*Mvh*), *Ar* (V. 1) and germline markers (**v**) 2.*Scp3*, 3.*ckit*, 4.*Integrin-αV*, 5.C*d9*, 6.*Integrin-β1*, 7. *Integrin-a6, 8. Plzf*, 9. *Mvh*) after 3 passages. Western blot result confirmed this co-culture system was sensitive to DHT or bicalutamide stimulation, 1, DMSO; 2, 10nM DHT; 3, 10nM bicalutamide, *n* = 3 (**w**)

**Fig. 3:**
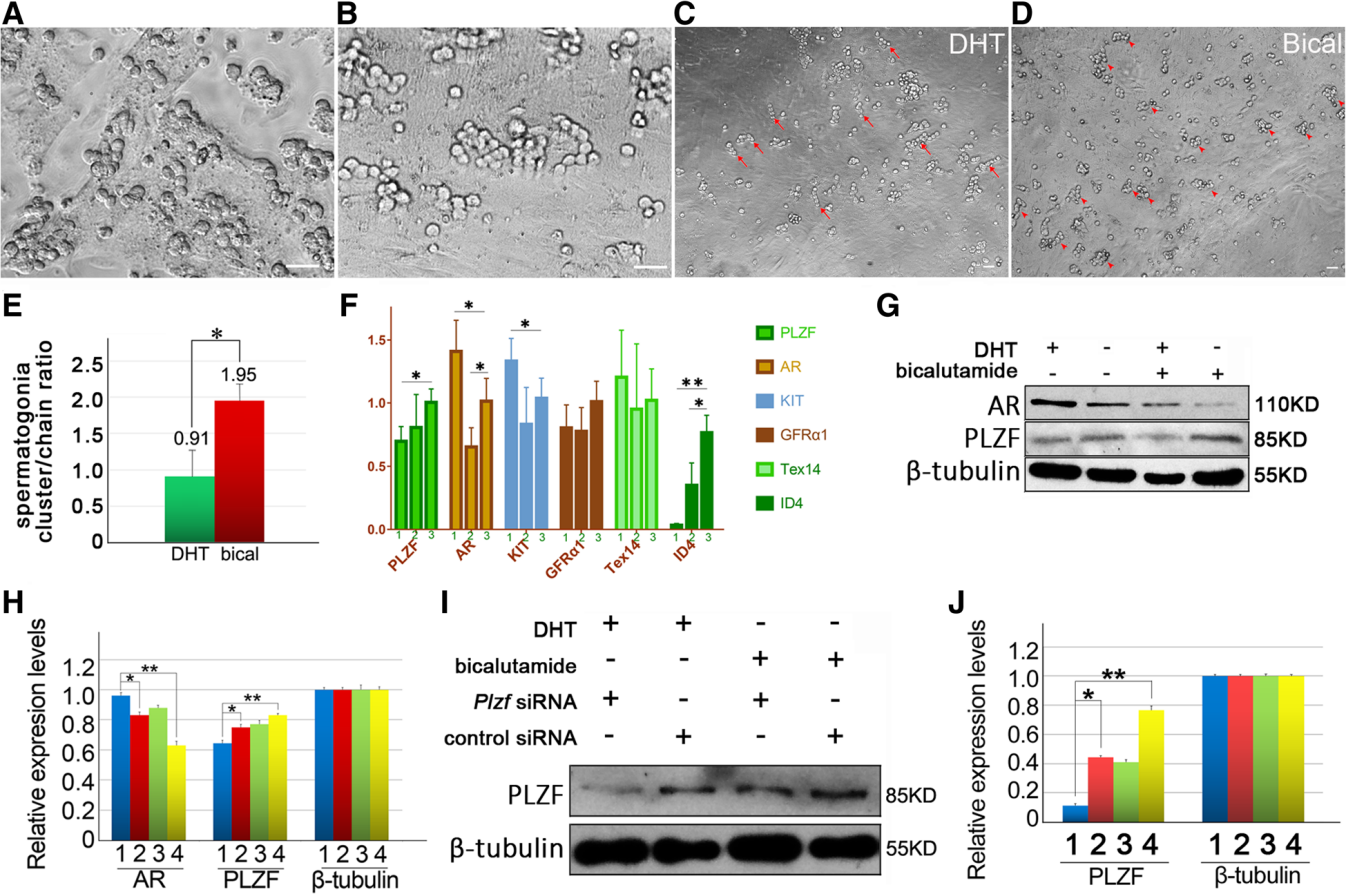
Impact of androgen on SPCs differentiation in SPCs-Sertoli cells co-culture system. Morphology of total testicular cells isolated from 5-day mice was displayed after 12 h’ culture (**a**). The morphology of co-culture system after two passages was exhibited (**b**). More A_al_ spermatogonia (red arrows) were observed in DHT treat group (**c**) while more clusters (red arrow heads) formed in bicalutamide treat group (**d**). The ratio of spermatogonia cluster/chain was calculated after DHT or bicalutamide treatment in co-culture system, *n* = 5 (**e**). Expression levels of *Plzf*, *Ar*, *c-kit*, *Gfrα1*, *Tex14* and *Id4* in co-culture system after DHT and/or bicalutamide treatment were evaluated by qRT-PCR, *n* = 4, and relative expression of indicated genes are quantitatively analyzed after normalization to *Gapdh.* 1, DHT; 2, bicalutamide; 3, DMSO (**f**). Impact on expression of AR and PLZF in co-culture system after DHT and bicalutamide treatment was determined by western blot, *n* = 6 (**g**), and the relative expression levels of AR and PLZF were statistically analyzed. 1, DHT+/bicalutamide-; 2, DHT-/bicalutamide-, 3, DHT+/bicalutamide+; 4, DHT-/bicalutamide+  (**h**). DHT aggravated the decline of PLZF expression caused by PLZF siRNA, while bicalutamide alleviated the knockdown effect, *n* = 5 (**i**), and the relative expression levels of PLZF were statistically analyzed, 1, DHT+/*Plzf *siRNA+; 2, DHT+/control siRNA+; 3, bicalutamide+/*Plzf *siRNA+; 4, bicalutamide+/control siRNA+. (**j**). Data represent means ± SD (*p < 0.05, **p < 0.01). Bar = 20 μM

To investigate the role of androgen in spermatogenesis, a co-culture system of testicular cells was established. Testes from 5 dpp mice (this time spermatogenesis just begins) were harvested and digested to single cells (composed of SPCs, differentiating spermatogonia and testicular somatic cells, and the markers of Sertoli cells (*Sox9* and *Wt-1*), Leydig cells (*Coup-tf II*) and Peritubular Myoid cells (*Sma*) were detected using RT-PCR (Fig. 2u)) and cultured on dishes. Somatic cells attached to gelatin and formed flat layers within 12 h’ culture, and undifferentiated spermatogonia population including SPCs attached on these layers [Fig. 3a]. This total testicular cells co-culture system can be maintained in vitro for 4 passages (16 days) at least [Fig. 3b]. Moreover, this co-culture system stably expressed germ line markers and somatic markers [Fig. 2a-v], and was sensitive to DHT or bicalutamide (an efficient AR antagonist) stimulation [Fig. 2w], indicating spermatogonia (including SPCs) and Sertoli cells were well maintained in this system and can be used to mimic spermatogenesis in vitro. To study androgen’s function in spermatogenesis, DHT was supplied to this co-culture system, and 2 h later bicalutamide was supplied to corresponding samples. After 48-h incubation, more A_al_ spermatogonia were observed in DHT treated group while more SPCs clusters were remained in bicalutamide treated group [Fig. 3a-e]. RT-PCR revealed DHT stimulation caused decreased expression levels of *Id4* and *Plzf* [Fig. 3f], indicating reduced size of undifferentiated spermatogonia populations including A_s_, A_pr_ and A_al_, while expression level of c-kit was increased [Fig. 3f]. Notably, expression of *Tex14*, a marker of intercellular bridge existing in both mitotic and meiotic divisions, namely from A_pr_, A_al_ spermatogonia, to spermatocytes [45], was up-regulated after DHT treatment [Fig. 3f]. These observations indicated that DHT reduced the population size of undifferentiated spermatogonia in this in vitro system. Results from western blot revealed expression levels of AR displayed opposite responses to those of PLZF after DHT/bicalutamide treatment [Fig. 3g and h]. Additionally, we observed that DHT aggravated the down-regulation of PLZF expression induced by *Plzf* siRNA [Fig. 3i and j], confirming that DHT promoted SPCs differentiation. On the contrary, Thy1-MACS purified SPCs cultured on gelatin or mitotically inactivated STO feeder layer (negative of AR expression) showed no obvious phenotype change after either DHT or bicalutamide treatment (data is not shown), suggesting that Sertoli cells were necessary for SPCs differentiation during androgen stimulation.

### AR indirectly regulates *Wt1* in Sertoli cells

**Fig. 4:**
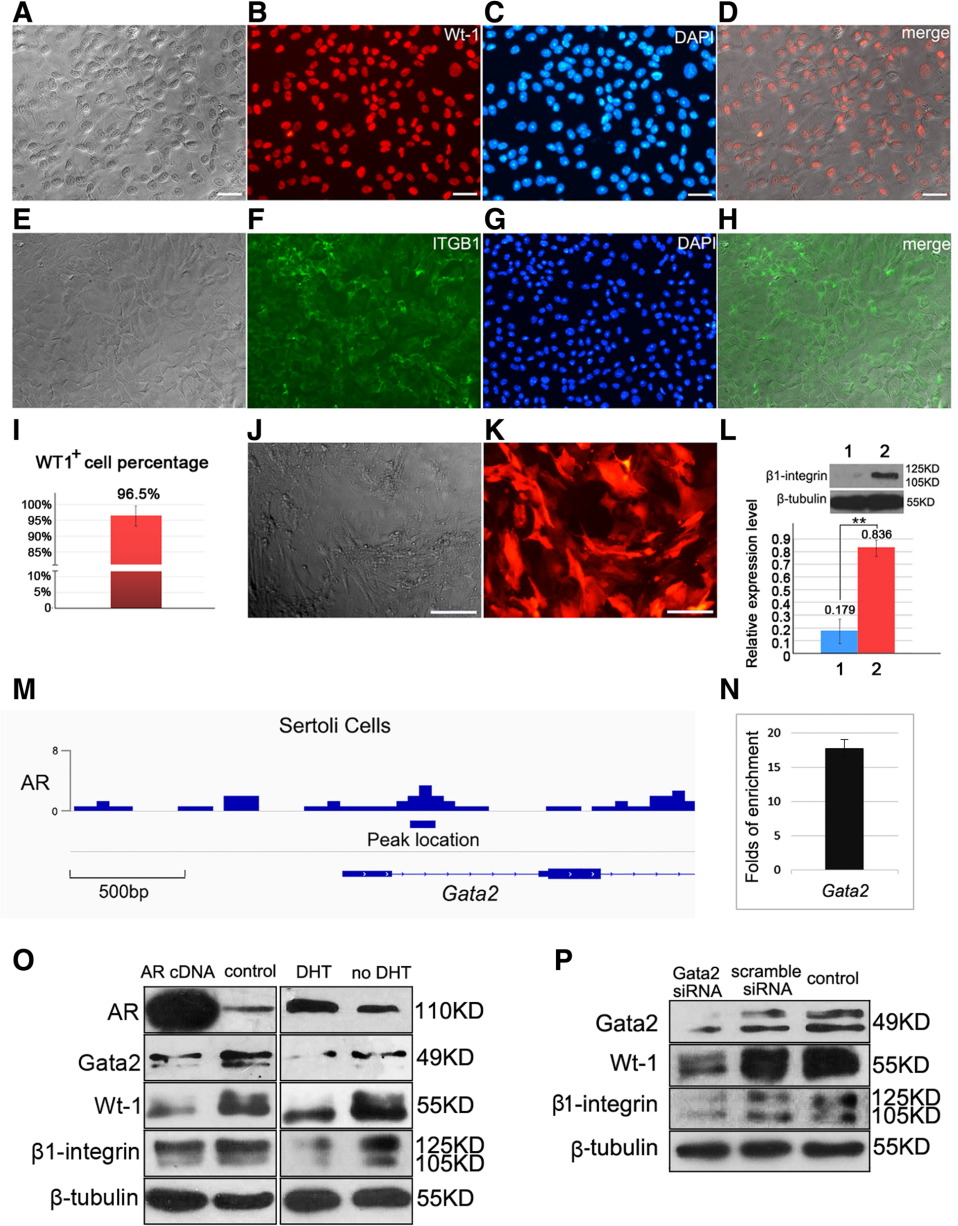
Purified Sertoli cells were used for co-culture and ChIP-seq analysis to screen target genes of AR in Sertoli cells. Bright field (**a**), WT1 staining (**b**), DAPI (**c**) and merge (**d**) of purified Sertoli cells were exhibited. WT1^+^ ratio of purified Sertoli cells was higher than 95%, *n* = 5 (**i**). Purified Sertoli cells were examined for β1-integrin expression by IF (**e-h**), and then were infected with β1-integrin shRNA lentivirus to interfere in endogenous expression of β1-integrin before co-culture with SPCs. Morphology of Sertoli cells 72 h post infection (**j**), and infection efficiency was examined by expression of red fluorescence protein reporter (**k**). Western blot revealed the relative expression level of β1-integrin was approximately decreased by 80% after β1-integrin shRNA interfering, 1, *Itgb1 *siRNA, 2, control siRNA. Data represent means ± SD (*p < 0.05, **p < 0.01), *n* = 3 (**l**). The representative view of AR binding sites (visualized by Integrative Genomics Viewer [http://software.broadinstitute.org/software/igv/]) at *Gata2* promoter region (**m**). ChIP-qPCR validated the binding of AR in the promoter region of *Gata2*: fold enrichment by antibodies against AR relative to control IgG was presented as mean value ± SD of two replicates (**n**). Overexpression of AR or DHT treatment caused down-regulated expression levels of GATA2, WT1 and β1-integrin in Sertoli cells, *n* = 3 (**o**). Knockdown of *Gata2* using siRNA led to decreased expressions of WT1 and β1-integrin, *n* = 3 (**p**)

To reveal regulatory mechanism of AR on spermatogenesis, we used ChIP-seq to screen the target genes of AR in purified Sertoli cells from 5 dpp mouse testis. In the candidates of targets, we noticed that *Gata2* promoter was a binding region of AR (*p*-value < 0.01 by MACS [36]) [Fig. 4m]. This result provided an important cue, since Gata2 is identified as a factor to promote WT1 expression by binding the enhancer of *Wt1* [46], and WT1 is a pivotal transcription factor for spermatogenesis in Sertoli cells [47]. Then the binding of AR on *Gata2* promoter region were repeatedly validated via ChIP-qPCR assays [Fig. 4n]. Furthermore, we observed that AR negatively regulated expression of Gata2 and WT1 after DHT stimulation or AR overexpression in Sertoli cells [Fig. 4o]. Consistently, knockdown of Gata2 caused down-regulated WT1 expression [Fig. 4p]. Taken together, these results implied that AR indirectly regulates expression of *Wt1* via *Gata2*.

### The *β1-integrin* gene is a direct target of WT1 in Sertoli cells

**Fig. 5:**
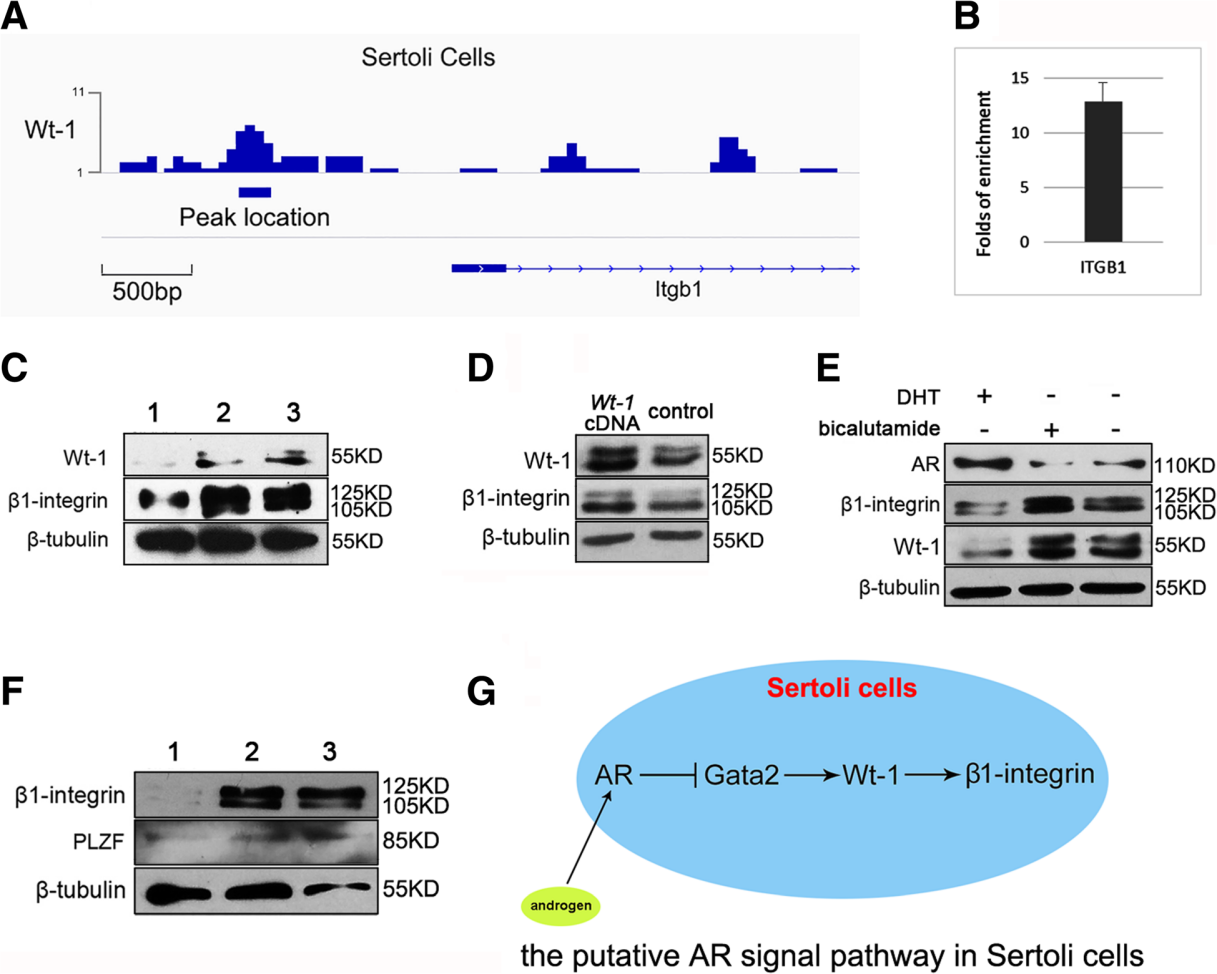
WT1 is an intermediate molecule to transmit androgen signal to downstream target β1-integrin. The representative view of WT1 binding sites (visualized by Integrative Genomics Viewer [http://software.broadinstitute.org/software/igv/]) at *β1-integrin* promoter region (**a**). ChIP-qPCR validates the binding of WT1 in the promoter region of *β1-integrin*: fold enrichment by antibodies against WT1 relative to control IgG was presented as mean value ± SD of two replicates (**b**). Knockdown of WT1 in Sertoli cells decreased β1-integrin expression, 1, *Wt-1* siRNA; 2, scambled siRNA; 3, untransfected control, *n* = 5 (**c**), and transfection of *Wt1* cDNA enhanced β1-integrin expression, *n* = 3 (**d**). Androgen down-regulated expression of WT1 and β1-integrin in co-culture system, *n* = 4 (**e**). Knockdown of β1-integrin in the co-culture system suppressed expression level of PLZF, 1, *Itgb1 *siRNA; 2, scambled siRNA; 3, untransfected control *n* = 3 (**f**). An illustration hypothesizes the potential signal pathway from AR to β1-integrin in Sertoli cells (**g**)

Since WT1 involves in multiple functions in Sertoli cells, we used ChIP-seq again to screen the target genes of WT1 regarding spermatogenesis in Sertoli cells. Notably, β1-integrin gene was identified as a direct binding target of WT1 [Fig. 5a]. Besides the role as a subunit of laminin receptor, β1-integrin is essential for SSCs homing since deletion of β1-integrin in SSCs or in Sertoli cells impaired SSCs homing [24], indicating its signal function in SSCs niche. After repeatedly validation of the binding of WT1 on β1-integrin gene using ChIP-qPCR [Fig. 5b], we further examined whether WT1 regulates β1-integrin expression at protein level. Knockdown of WT1 expression in Sertoli cells suppressed expression of β1-integrin [Fig. 5c], and WT1 overexpression strengthened β1-integrin’s expression level [Fig. 5d], suggesting that expression of β1-integrin was regulated by WT1. Moreover, we observed that androgen signal negatively regulated the expression of β1-integrin in Sertoli cells [Fig. 5e], and disturbance of Gata2 expression displayed similar impact on β1-integrin expression [Fig. 4p], which were consistent with aforementioned results that AR negatively regulated Gata2 via binding on its promoter region, and indirectly interfered in WT1 expression. Then we knockdown the expression of β1-integrin in co-culture system using siRNA and observed decreased expression level of PLZF [Fig. 5f], implying the indispensable role of β1-integrin in SPCs maintenance. Collectively, we concluded a cue from androgen signal to surface protein β1-integrin, via intermediate factor GATA2 and WT1 [Fig. 5g].

### β1-integrin on Sertoli cells may function as an intermediate molecule to regulate SPCs differentiation

**Fig. 6:**
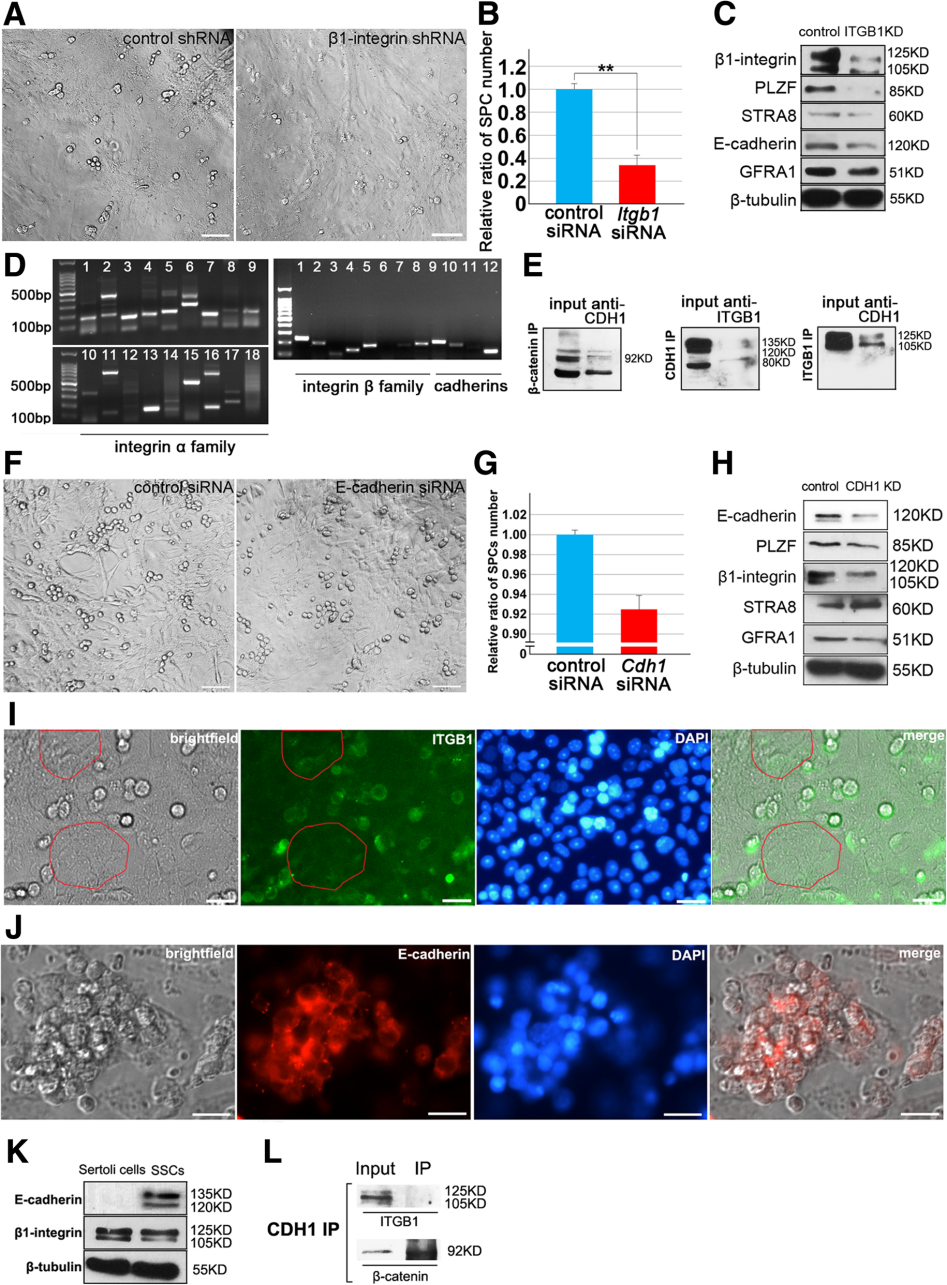
Identification of β1-integrin as a putative interactive molecule between SPCs and Sertoli cells. Thy1^+^ SPCs were co-cultured with Sertoli cells infected with control shRNA or β1-integrin shRNA for 48 h (**a**), bar = 20 μM, and the relative ratio of SPCs number of β1-integrin shRNA transfected group compared to control was statistically analyzed (**b**). The expression levels of germline markers of SPCs were detected using Western blot, *n* = 3 (**c**). RT-PCR detected expression profiles of integrins and cadherins in SPCs at mRNA level, (1-18 in integrin α family represent α1~α11,αD, αE, αL, αM, αV, α2b, αX; 1-12 in integrin β family and cadherins represent β1-β8, E-cadherin, N-cadherin,  P-cadherin and Gapdh, respectively) (**d**). The binding of E-cadherin and β1-integrin was detected in adult mouse testis cell lysates using immunoprecipitation, of which the binding of β-catenin and E-cadherin was used as positive control, *n* = 3 (**e**). Thy1^+^ SPCs co-cultured with Sertoli cells were transfected with control siRNA or E-cadherin siRNA for 48 h (**f**), bar = 20 μM, and the relative ratio of SPCs number was statistically analyzed (**g**). The expression levels of germline markers of SPCs were detected using Western blot, *n* = 3 (**h**). Expression of β1-integrin (**i**) and E-cadherin (**j**) in SPCs and co-cultured Sertoli cells was determined by IF, and β1-integrin signal was detected in Sertoli cells (red frames), bar = 20 μM. Purified Sertoli cells or Thy-1^+^ SPCs were lysed to detect the expression of β1-integrin and E-cadherin using western blot, *n* = 3 (**k**). Thy-1^+^ SPCs were lysed for co-IP to detect the endogenous interaction of β1-integrin and E-cadherin, *n* = 2 (**l**)

The important role of β1-integrin in vivo has been proven in Sertoli cells specific β1-integrin conditional knockout mice [24]. Here we used in vitro co-culture system to verify the role of β1-integrin on Sertoli cells for SPCs maintenance. Because primary Sertoli cells have limited proliferation capacity in vitro, and knockout of β1-integrin in Sertoli cells causes serve damage, primary Sertoli cells were infected with β1-integrin shRNA or control shRNA lentivirus for 72 h to attenuate β1-integrin expression, and were used as feeder layers for Thy1^+^ SPCs culture (Knockdown efficiency of β1-integrin was evaluated in Fig. 4j-l). After co-culture for 48 h, number of SPCs on β1-integrin disturbed Sertoli cells was significantly less than control group [Fig. 6a and b], and western blot confirmed that SPCs on β1-integrin disturbed feeder expressed lower levels of SPCs markers [Fig. 6c], indicating that β1-integrin on Sertoli cells affects the maintenance of SPCs in this co-culture system. Then we hypothesized that β1-integrin on Sertoli cells interacted with some molecules on SPCs to regulate their fates. To screen these putative molecules interacted, expression of integrins and cadherins in SPCs were examined using sqRT-PCR [Fig. 6d]. We focused on E-cadherin as a candidate since SPCs highly and specifically express E-cadherin. Subsequently, the binding of β1-integrin and E-cadherin was validated in adult mouse testis lysates using immunoprecipitation. E-cadherin was pulled down by the antibody against-β1-integrin, and vice versa [Fig. 6e], implying that E-cadherin on SPCs binds with β1-integrin in physiological condition. Moreover, E-cadherin siRNA was transfected into Thy1^+^ SPCs Sertoli cells co-culture system for 48 h, to detect the impact on SPCs fates. Unexpectedly, no obvious difference of growth condition or cell number was observed [Fig. 6f and g]. However, western blot revealed that E-cadherin knockdown reduced expression levels of SPCs markers, including PLZF, ITGB1, GFRα1, and up-regulated expression level of STRA8 [Fig. 6h], indicating that E-cadherin could interfere in SPCs at least at molecular level. Notably, E-cadherin is a specific undifferentiated spermatogonia marker, while β1-integrin is expressed on both spermatogonia and Sertoli cells [48, 49], which is consistent with our results from IF [Fig. 6i-j and Fig. 4e-h] and from western blot [Fig. 6k]. Thus, it’s not clear that E-cadherin on SPCs binds with β1-integrin on Sertoli cells or on SPCs themselves. Therefore, Thy1^+^ SPCs were used for IP [Fig. 6l], and the result eliminates the possibility that E-cadherin and β1-integrin on SPCs binds on themselves. These results suggest that β1-integrin on Sertoli cell is essential for SPCs maintenance, and probably interacts with E-cadherin on SPCs.

### Pharmacologically androgen deprived mice are lack of differentiated germ cells but enriched with hyper-proliferated PLZF^+^ and β1-integrin^+^ spermatogonia

**Fig. 7:**
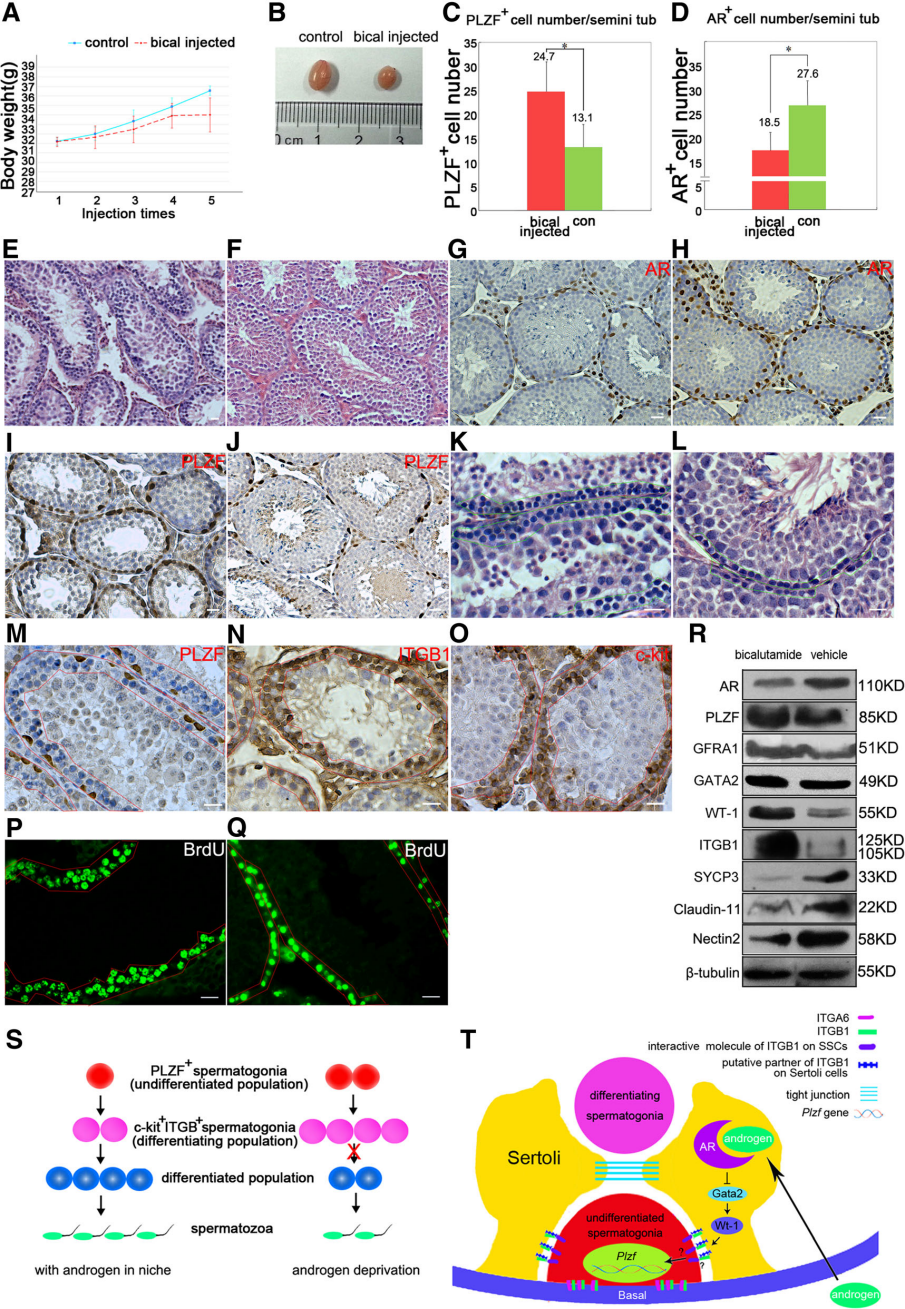
Bicalutamide recipient mice lack of spermatocytes and spermatids, but possess hyper-proliferative SPCs. Bicalutamide recipient mice (*n* = 33) exhibited retarded body weight growth (**a**) and smaller testis size (**b**). Histology results demonstrated that spermatocytes and spermatids in seminiferous tubules of bicalutamide recipients (**e**) were significantly reduced compared to controls (**f**), bar = 20 μM. IHC staining displayed reduced number of AR^+^ cells (**g**) and increased number of PLZF^+^ cells (**i**) in bicalutamide recipients than in controls (**h** and **j**). A statistical result indicated a augmented population of PLZF^+^ cells (**c**) and a remarkable decline of AR^+^ cell population (**d**) in seminiferous tubules of bicalutamide recipients, data represent means ± SD (*p < 0.05). Accumulated spermatogonia formed two layers in bicalutamide recipient seminiferous tubules (green frame in **k**), while wild type seminiferous tubule possessed only one layer of spermatogonia (green frame in **l**). Most of spermatogonia in those two layers were β1-integrin^+^ (**n**), c-kit^+^ (**o**) PLZF^−^ (**m**). BrdU assay indicated they were mitosis active (**p**), as those in wild type (**q**). Spermatogonia layers were enclosed by red frames in **m**, **n**, **o**, **p** and **q**. Bicalutamide recipient testes displayed down-regulated expression of AR, SYCP3, Claudin-11, WT1, Nectin-2, and up-regulated expression of PLZF, GFRA1, GATA2, WT1 and β1-integrin, *n* = 4 (**r**). Androgen deprivation may block spermatogenesis at the step that β1-integrin^+^c-kit^+^ population transits to more differentiated germ cells, causing accumulation of undifferentiated spermatogonia (**s**). Androgen produced by Leydig cells interacts with AR in Sertoli cells to regulate down-stream target genes, and subsequently transfer differentiation signals to SPCs, and finally turns off the differentiation inhibitor gene *Plzf* via multiple steps. *Gata2* was identified as a target of AR, and *Wt1* was activated by GATA2 to regulate downstream gene *β1-integrin* in Sertoli cells. How β1-integrin on Sertoli cells influences SPCs’ fates via regulating *Plzf* is unknown (**t**)

To further study the role of androgen on spermatogenesis, 33 6-week old male mice were intraperitoneally injected with bicalutamide, since mice at this age have mature Sertoli cells, stable AR expression and continuous spermatogenesis. Bicalutamide recipient mice exhibited retarded body weight growth and smaller testis size compared to control group [Fig. 7a **and** b]. Histological results revealed the severe deficiency of differentiated germ cells (spermatocytes, spermatids and sperms) in seminiferous tubules [Fig. 7e]. Moreover, number of AR^+^ cells [Fig. 7d] and expression level of AR [Fig. 7g and r] were reduced, but number of PLZF^+^ cells [Fig. 7c] and expression level of PLZF [Fig. 7i and r] were increased in bicalutamide recipient testes compared to controls [Fig. 7h and j], indicating that androgen deprivation did not impair undifferentiated spermatogonia, but resulted in blockage of germ cell differentiation and expansion of undifferentiated spermatogonia population. Notably, in many seminiferous tubules of bicalutamide recipients, accumulated spermatogonia formed a second layer [Fig. 7k], which were identified as PLZF negative [Fig. 7m], β1-integrin and c-kit positive [Fig. 7n and o], and mitotically active [Fig. 7p] spermatogonia. These results indicated androgen deprivation blocked spermatogenesis at the step that ckit^+^β1-integrin^+^ spermatogonia differentiate to more differentiated populations, and consequently led to accumulation of undifferentiated spermatogonia, including PLZF^+^ population [Fig. 7s]. Western blot results showed the increased expressions of Gata2, WT1 in bicalutamide recipients [Fig. 7r], which was consistent with results from in vitro. Enhanced expression levels of PLZF, GFRA1 and β1-integrin resulted from accumulated spermatogonia, and decreased expression levels of SYCP3, Claudin-11 and Nectin-2 were the consequence of lost meiotic germ cells caused by androgen deprivation [Fig. 7r]. These results implied that blockage of androgen in adult mouse testis inhibited the differentiation from spermatogonia to spermatocytes, and meanwhile promoted SPCs accumulation.

## Discussion

Our results exhibit that SPCs are deficient of endogenous AR after 3 dpp, which is consistent with the dispensable role of AR in differentiated germ cells from AR germ cell conditional knockout mice. Interestingly, some studies detected AR expression in germ cells of fetal testis [50], and we examined weak AR signal in pre-spermatogonia of 2 dpp old testes. Therefore, we prefer the opinion that SPCs do not need endogenous AR for the initiation of spermatogenesis after neonatal stage, and the function of AR in embryonic and new born stages is still need to be revealed.

Testis Zinc Finger Protein (TZFP), a homolog of PLZF, could co-repress the activated AR [51] and participate in hormone signal pathway to regulate spermatogenesis [52]. However, IHC staining showed PLZF and AR staining were distributed in distinct cell types after neonatal stage, indicating no direct interaction between these two transcription factors during spermatogenesis. Based on our data, we hypothesize that transduction of androgen signal in spermatogenesis spatially can be summarized into three steps [Fig. 7t]: the first step is AR activation in Sertoli cells induced by androgen, including androgen-AR interaction and activation of AR’s downstream genes to produce signal molecules; second step is the communication between Sertoli cells and SPCs, involving in some surface or transmembrane proteins; the last one is signal transduction to SPCs nucleus to turn off PLZF and start differentiation. Our results suggest that AR inhibits Gata2 expression in Sertoli cells by binding on its promoter region, leading to a consequent decrease of WT1 expression, and WT1 binds to and regulates β1-integrin in Sertoli cells, which interacts with SPCs as a putative signal molecule to regulate SPCs differentiation. WT1 is able to bind the promoter of *Ar* to inhibit AR expression [28]. We revealed AR’s inhibitory role on WT1 expression via interacting with Gata2, indicating a feedback regulation on WT1 expression. Moreover, we proposed β1-integrin on Sertoli cells regulates SPCs differentiation. Routinely, β1-integrin combines with another subunit, i.e. α1-integrin, α6-integrin, to form a complex. On SPCs surface β1-integrin binds α6-integrin to form laminin receptor, which is essential for SPCs maintenance. Nevertheless, it’s not clear that α6-integrin binds with β1-integrin in Sertoli cells [53], thus we are interesting in searching for the interactive molecule of β1-integrin on Sertoli cells in future study. Moreover, it’s not clear E-cadherin really functions as a signal molecule to regulate SPCs fates in responding to β1-integrin on Sertoli cells, since loss of E-cadherin in SPCs did not impair homing capacity. Our data also exhibited that the growth condition and cell number were unaffected after E-cadherin knockdown (Fig. 6f and g). However, we cannot conclude E-cadherin is redundant for SSCs, since SSCs enrich other types of cadherins which may compensate the role of E-cadherin, and some studies observed that E-cadherin mutation in SSCs led slightly reduced colony formation after transplantation [24]. Notably, our observations revealed the reduced expression levels of E-cadherin do have impact on expression levels of SPCs markers (Fig. 6h). A possibility is that the time for E-cadherin siRNA treatment is not long enough. Although other cadherins in SPCs may compensate the function of E-cadherin, loss of E-cadherin may regulate its own downstream targets or interactive molecules, which will impact on the fates of SPCs. For example, E-cadherin was reported to be functional in transformation of SSCs into pluripotent status [54]. Thus, next we will focus on the link of E-cadherin and SSCs fates, especially regarding β1-integrin’s binding partners on SSCs surface.

Two important junction proteins Clauding-11 and Nectin-2, which were reported to be significantly affected in testes of AR knockout mouse [25], declined in bicalutamide recipient testes. Claudin-11 is a key component of tight junction (TJ) barrier and closely related to spermatogenesis, and its deficiency caused infertility [55]. *Ar* deletion in Sertoli cells resulted in down-regulation of Claudin-3 (a homolog of Claudin-11 and a component of TJ junction) and a subsequent increase of BTB (blood-testis barrier) permeability [56]. Further study revealed that Claudin-11 replaced Claudin-3 during spermatocyte translocation in spermatogenesis [57]. Therefore, we inferred that androgen deprivation caused the blockage of spermatogonia differentiation and a consequent accumulation of SPCs. Reduced Claudin-11 expression after androgen deprivation indicated loose of TJ junction. Whether Claudin-11 interacts with β1-integrin is also an interesting question. Down-regulation of Nectin-2 after androgen deprivation from our experiments exhibited contradicting result to that from AR knockout mice [25]. As a anchor junction protein, Nectin-2 is mainly expressed in Sertoli cells and elongated spermatids, and *Nectin-2* knockout mice had normal sperm titers but exhibited infertility phenotype due to serve spermatozoa malformation [58], suggesting the major role of Nectin-2 is for sperm mature. Thus, the dramatic decline of Nectin-2 expression in androgen deprivation assay was due to blockage of spermatogonia differentiation into spermatocytes and spermatids.

## Conclusions

This study reveals androgen signal pathway in Sertoli cells which promotes differentiation of PLZF+ spermatogonia. GATA2, WT1 and β1-integrin were identified as pivotal intermediate molecules in this process. The take-home messages are listed below

1. The expression profile of androgen receptor (AR) in testes of postnatal male mice reveals that spermatogonia express AR before homing to their niche at 2 dpp, but do not express AR since spermatogenesis starts,
2. Sertoli cells are required for spermatogonia differentiation verified by a spermatogonia-Sertoli cells co-culture system.
3. Data from ChIP-seq and gene silence or overexpression assays conclude that AR is able to regulate WT-1 (a key factor for Sertoli cells) indirectly, and identify β1-integrin (a key surface molecule regulating SPCs homing) as a direct target of WT-1in Sertoli cells,
4. Pharmacologically androgen deprived mice process accumulated undifferentiated spermatogonia and differentiating spermatogonia, but lack spermatocytes and sperm in testes.

In the future, we will focus on the communication pattern between Sertoli cells and SPCs, in particular β1-integrin’s role in regulation of SSCs fates.

## Additional file

Additional file 1: **Table S1.** primer information for semi-quantitative and quantitative RT-PCR. (DOCX 28 kb)

## Abbreviations

AR: Androgen receptor
Coup-tf II: Chicken ovalbumin upstream promoter-transcription factor
DHT: Dihydrotestosterone
dpp: Day post partum
GFRα1: Glial cell line derived neurotrophic factor family receptor alpha 1
ID4: Inhibitor of DNA binding 4
Mvh: Mouse vasa homologue
PLZF: Promyelocytic leukemia zinc-finger
SMA: A-smooth muscle actin
SPCs: Spermatogonia progenitor cells
SSCs: Spermatogonial stem cells
STO cells: SIM mouse embryo-derived thioguanine-and ouabain-resistant cells
STRA8: Stimulated by retinoic acid gene 8
SYCP3: Synaptonemal complex protein 3
TEX14: Testis expressed gene 14
WT1: Wilms’ tumor 1
