# Supplementary material for: Androgen promotes differentiation of PLZF+ spermatogonia pool via indirect regulatory pattern
Source: Cell Commun Signal. 2019 May 29;17:57. doi: 10.1186/s12964-019-0369-8 (PMC6542041; doi:10.1186/s12964-019-0369-8)
Supplement: Supplementary file 1 — Table S1. primer information for semi-quantitative and quantitative RT-PCR. (DOCX 28 kb) [file 12964_2019_369_MOESM1_ESM.docx]

Additional file 1: **Table S1.** primer information for semi-quantitative and quantitative RT-PCR

| primer sequences for semi-quantitative RT-PCR (5’-3’) | | | |
| --- | --- | --- | --- |
| Itga1 | F:GGGCAGCCTCGGTACAATCA | Itgb2 | F:GGTATGACGCTGCAGACTATCC |
|  | R:AGCCCCGACGAGAAGCAGAT |  | R:CAGTACGACACCTACCACGG |
| Itga2 | F:CCATGATGGGTCGAAGCTGA | Itgb3 | F:TGGCAAGTACTGTGAGTGCG |
|  | R:CTTCGTCGGCCACATTGAAA |  | R:TCCAGTCCGAGTCACACACG |
| Itga3 | F:CAACATTACCAATGTGACCGTGAAA | Itgb4 | F:GCCTACGAGGTCTGCTATGG |
|  | R:ACCATGTGGTCTTGTTCTCCATGTT |  | R:CGCCTTAACCGTGTATCGG |
| Itga4 | F:TGTGGAAGGCTGGATTCTTT | Itgb5 | F:GCCAAGATGGCATATCTTACC |
|  | R:CGGGTCTTCTGAACAGGATT |  | R:TGCAATTGTAGGCGACTTCC |
| Itga5 | F:CAACCCAGAGGAGCGCAGTT | Itgb6 | F:TGGAATATCCAACTATCGGCC |
|  | R:TAAGGGTGGCCTGCTTGGAA |  | R:ACCGCAGTTCTTCATAAGCG |
| Itga6 | F:GAGGAATATTCCAAACTGAACTAC | Itgb7 | F:GAGGTCACACATTCTGTGCG |
|  | R:GGAATGCTGTCATCGTACCTAGAG |  | R:TCTCTCTCGAAGGCTTGAGC |
| Itga7 | F:CTCTCCCAGCCTCTCTACGGTACTC | Itgb8 | F:AAGGATCCACAATCAGTGC |
|  | R:CAGAGGTGCTGAGGATGAGGTAAAA |  | R:CCAATATGACTCTCACAGACG |
| Itga8 | F:CGAGGTGCAGTTAGATTTCCTGAAG | Cdh1 | F:ACCGATTCAAGAAGCTGGC |
|  | R:TGTAGTTCAGGCTGATGTTGATTGG |  | R:ACCATCCTAACACAGACAGTCC |
| Itga9 | F:GAAAGGAATTGCCAATCTGAGGACT | Cdh2 | F:CATGCTGAGCCACAGTACC |
|  | R:CCTGGAGACATTAAAGGACACGTTG |  | R:CGCTACTGGAGGAGTTGAGG |
| Itga10 | F:CTCTGCTCCTCCTTTAATCTGGATG | Cdh3 | F:GGACCAGGACTATGACATCACC |
|  | R:GAGCAACGATAAACATCCCCTCTCC |  | R:TGTTGGCAGCCTTCAGG |
| Itga11 | F:GGAGTTTCCAGAGGAGCTGAAGAAC | WT1 | F:GTGAAACCATTCCAGTGTAAAAC |
|  | R:AGAATGACCTTGCCAGTATGGTTGA |  | R:GCCACCGACAGCTGAAGGGC |
| ItgaD | F:AACCTGGATGTGGAGAAGCCCG | SOX9 | F:GCCGACTCCCCACATTCCTCC |
|  | R:GACCACAAGCCAGCAACTGGG |  | R:GCCGTAACTGCCAGTGTAGGTG |
| ItgaE | F:GGACGATCAAGCAACATCAA | Acta2 | F:GAGAAGCCCAGCCAGTCG |
|  | R:GGAACCGTGCTCATTAAAGG |  | R:CTCTTGCTCTGGGCTTCA |
| ItgaL | F:TTGAGGGCACAAACAGACAG | Nr2f2 | F:AAGAGCTTCTTCAAGCGCAG |
|  | R:TCATCCAGGCCACAGTGTAA |  | R:CCTCTCTGTACAGCTTCCCG |
| ItgaM | F:CAGATCAACAATGTGACCGTATGG | MVH | F:GGAAACCAGCAGCAAGTGAT |
|  | R:CATCATGTCCTTGTACTGCCGC |  | R:TGGAGTCCTCATCCTCTGG |
| ItgaV | F:TCTATATTGGGGACGACAACCCTCT | AR | F:GGACCATGTTTTACCCATCG |
|  | R:AATGCACAGGACAGTCTTGCTAAGG |  | R:TCGTTTCTGCTGGCACATAG |
| Itga2b | F:GTGGGGAAGACGACCTGTGTG | SYCP3 | F:TGAGTCTTTGAAGAAAGAACTTGA |
|  | R:CAAGCCTCTCAAAGCCCTCAAT |  | R:AAATTTAAATCATCTTTATTGACACA |
| ItgaX | F:ACACAGTGTGCTCCAGTATGA | c-Kit | F:TCATCGAGTGTGATGGGAAA |
|  | R:GCCCAGGGATATGTTCACAGC |  | R:GGTGACTTGTTTCAGGCACA |
| Itgb1 | F:GTCTGTTTGCAATATGGGGG | CD9 | F:ATCTTCTGGCTCGCTGGCATT |
|  | R:GCACTGTCAAAATGAAAAGGC |  | R:ATGGCTTTGAGTGTTTCCCGCT |
| Itgb2 | F:GGTATGACGCTGCAGACTATCC | GAPDH | F:CCTGGAGAAACCTGCCAAGTATG |
|  | R:CAGTACGACACCTACCACGG |  | R:AGAGTGGGAGTTGCTGTTGAAGTC |
| primer sequences for quantitative RT-PCR (5’-3’) | | | |
| AR | F:CTGGGAAGGGTCTACCCAC | PLZF | F:CACACTCAAGAGCCACAAGC |
|  | R:GGTGCTATGTTAGCGGCCTC |  | R:ATCATGGCCGAGTAGTCTCG |
| c-Kit | F:GCCACGTCTCAGCCATCTG | GFRα1 | F:TACCACCAGCATGTCCAATGAA |
|  | R:GTCGGGATCAATGCACGTCA |  | R:GTAGCTGTGCTTGGCTGGAACT |
| TEX14 | F:GTTTACGAGCGTATCACAGTCG | ID4 | F:GAGACTCACCCTGCTTTGCT |
|  | R:TATATCAAGGCATCAGCAACCT |  | R:ATGCTGTCACCCTGCTTGTT |
|  |  | GAPDH | F:CCTGGAGAAACCTGCCAAGTATG |
|  |  |  | R:AGAGTGGGAGTTGCTGTTGAAGTC |
